# Determination of reference genes for circadian studies in different tissues and mouse strains

**DOI:** 10.1186/1471-2199-11-60

**Published:** 2010-08-16

**Authors:** Rok Kosir, Jure Acimovic, Marko Golicnik, Martina Perse, Gregor Majdic, Martina Fink, Damjana Rozman

**Affiliations:** 1 Center for Functional Genomics and Bio-Chips, Institute of Biochemistry, Faculty of Medicine, University of Ljubljana, Zaloska 4, SI-1000 Ljubljana, Slovenia; 2 Institute of Biochemistry, Faculty of Medicine, University of Ljubljana, Vrazov trg 2, SI-1000 Ljubljana, Slovenia; 3 Medical Experimental Centre, Institute of Pathology, Faculty of Medicine, University of Ljubljana, Zaloska 4, SI-1000 Ljubljana, Slovenia; 4 Center for Animal Genomics, Veterinary Faculty; University of Ljubljana, Gerbiceva 60, SI-1000 Ljubljana, Slovenia; 5 University Medical Center Ljubljana, Department of Haematology, Zaloska cesta 7, SI-1000 Ljubljana, Slovenia

## Abstract

**Background:**

Circadian rhythms have a profound effect on human health. Their disruption can lead to serious pathologies, such as cancer and obesity. Gene expression studies in these pathologies are often studied in different mouse strains by quantitative real time polymerase chain reaction (qPCR). Selection of reference genes is a crucial step of qPCR experiments. Recent studies show that reference gene stability can vary between species and tissues, but none has taken circadian experiments into consideration.

**Results:**

In the present study the expression of ten candidate reference genes (*Actb*, *Eif2a*, *Gapdh*, *Hmbs*, *Hprt1*, *Ppib*, *Rn18s*, *Rplp0*, *Tbcc *and *Utp6c*) was measured in 131 liver and 97 adrenal gland samples taken from three mouse strains (C57BL/6JOlaHsd, 129Pas plus C57BL/6J and *Crem *KO on 129Pas plus C57BL/6J background) every 4 h in a 24 h period. Expression stability was evaluated by geNorm and NormFinder programs. Differences in ranking of the most stable reference genes were observed both between individual mouse strains as well as between tissues within each mouse strain. We show that selection of reference gene (*Actb*) that is often used for analyses in individual mouse strains leads to errors if used for normalization when different mouse strains are compared. We identified alternative reference genes that are stable in these comparisons.

**Conclusions:**

Genetic background and circadian time influence the expression stability of reference genes. Differences between mouse strains and tissues should be taken into consideration to avoid false interpretations. We show that the use of a single reference gene can lead to false biological conclusions. This manuscript provides a useful reference point for researchers that search for stable reference genes in the field of circadian biology.

## Background

Circadian rhythms are oscillations in behaviour and physiology whose function it is to anticipate environmental changes associated with the solar day [1]. At the molecular level, they consist of a network of transcriptional and translational feedback loops that drive the 24 h expression of core clock components [2,3]. Circadian control is required for healthy life, thus disruption of circadian cycle leads to pathologies such as cancer, obesity, lipid disorders and type 2-diabetes [4-6]. Some of these abnormalities were discovered in mouse models lacking core clock genes *Clock *and *Bmal1 *[7-9]. Phenotypes resulting from mutations of clock genes are highly affected by genetic background [4,10,11]. Yoshiki defined genetic background as the influence of all genes of the genome that may interact with the gene of interest and potentially influence the specific phenotype [12]. To date, a number of reports have shown that genetic background affects the phenotype caused by gene disruption [12-15].

When studying gene expression, qPCR is the dominant quantitative technique due to its broad dynamic range, accuracy, sensitivity, specificity and speed [16]. Normalization in qPCR controls for variations in all experimental steps, enabling comparison between different samples [17]. Different normalization strategies are available, where application of reference genes as internal controls seems to be the most appropriate [18,19]. Unfortunately, there is no universal reference gene that would be stably expressed under all experimental conditions. Hence, normalization to reference genes that are validated in individual experiments is a prerequisite for accurate interpretation of biological data [20-24].

The broader scope of our research is to understand the circadian expression of genes in different tissues of the two commonly used laboratory mouse strains (C57BL/6JOlaHsd and 129Pas plus C57BL/6J) and to determine the tissue-specific effect of the targeted disruption of transcription factor *Crem*. In this manuscript, we show that genetic background and the circadian time are important factors influencing expression of commonly used qPCR reference genes. This should be taken into consideration for accurate interpretation of biological data.

## Results

### Selection and characteristics of candidate reference genes

Seven candidate reference genes (*Ppib*, *Rplp0*, *Gapdh*, *Actb*, *Hmbs*, *Hprt1 *and *Rn18s*) were selected [20,23] and their expression measured in 35 livers and 33 adrenals from the inbred strain (C57BL/6JOlaHsd), 51 livers and 34 adrenals from the wild type mixed strain (129Pas plus C57BL/6J) and 45 livers and 30 adrenals from the mixed strain with a targeted disruption of the *Crem *gene (*Crem *KO). For livers, three additional reference genes were selected by RefGenes (*Eif2a*, *Utp6c *and *Tbcc*), based on meta analysis of the most stably expressed liver genes in various mouse strains as detected by Affymetrix chips [25]. Gene symbols, full names, accession numbers and gene functions are listed in Table 1. Intron spanning primers were designed wherever possible, with the exception of *Rplp0 *and *Tbcc *where primers lie within a single exon. To determine primer specificity, melting curve analyses were performed on all primer pairs during the primer validation process as well as after each qPCR run. The specificity of the amplicon was confirmed by the presence of a single peak. Primer efficiencies were calculated based on slopes from standard curves by LightCycler 480 software (Roche Diagnostics). Standard curves were prepared with five-fold serial dilutions of the cDNA pool. A negative control (without reverse transcriptase) was also included to determine possible amplification from genomic DNA. Only primers with single peaks and good negative controls were used in the study. Primer details are listed in Table 2.

**Table 1:** Official symbols, accession numbers, full names and functions of candidate reference genes evaluated in this study.

Symbol	Accession number	Full name	Function
*Rplp0*	NM_007475.4	ribosomal protein, large, P0	Structural constituent of ribosome
*Ppib*	NM_011149.2	peptidylprolyl isomerase B	Associated with the secretory pathway and released in biological fluids
*Actb*	NM_007393.3	actin, beta	Cytoskeletal structural protein
*Hmbs*	NM_013551.2	Hydroxymethyl-bilane synthase	Heme synthesis, porphyrin metabolism
*Hprt1*	NM_013556.2	hypoxanthine guanine phosphoribosyl transferase 1	Purine synthesis in salvage pathway
*Rn18s*	NR_003278.1	18 S RNA	Ribosomal RNA
*Eif2a*	NM_001005509.1	eukaryotic translation initiation factor 2a	Protein translation
*Utp6c*	NM_144826.3	small subunit (SSU) processome component, homolog (yeast)	Rn18 s biogenesis
*Tbcc*	NM_178385.3	tubulin-specific chaperone c	Protein folding

**Table 2:** Primer sequences, exon location (where possible), efficiency and amplicon length for candidate reference genes and the test circadian gene.

Symbol	Primer	Sequence	Amplicon length	Primer location^1 ^[exon]	Primer efficiency
**Reference genes**					
				
*Rplp0*	fw	CACTGGTCTAGGACCCGAGAAG	73	4	1.98
	rv	GGTGCCTCTGGAGATTTTCG		4	
*Ppib*	fw	GGAGATGGCACAGGAGGAAA	73	3	1.93
	rv	CCGTAGTGCTTCAGTTTGAAGTTCT		4	
*Gapdh*	fw	CCAATGTGTCCGTCGTGGATCT	239	5	1.94
	rv	GTTGAAGTCGCAGGAGACAACC		6	
*Actb*	fw	CTTCCTCCCTGGAGAAGAGC	124	4	1.98
	rv	ATGCCACAGGATTCCATACC		5	
*Hmbs*	fw	TCCCTGAAGGATGTGCCTA	73	7	1.64
	rv	AAGGGTTTTCCCGTTTGC		8	
*Hprt1*	fw	TCCTCCTCAGACCGCTTTT	90	1	1.89
	rv	CCTGGTTCATCATCGCTAATC		2	
*Rn18s*	fw	CGCCGCTAGAGGTGAAATTC	62	n.a.	1.79
	rv	TTGGCAAATGCTTTCGCTC		n.a.	
*Eif2a*	fw	CAACGTGGCAGCCTTACA	74	2	1.95
	rv	TTTCATGTCATAAAGTTGTAGGTTAGG		3	
*Utp6c*	fw	TTTCGGTTGAGTTTTTCAGGA	75	17	1.82
	rv	CCCTCAGGTTTACCATCTTGC		18	
*Tbcc*	fw	GACTCCTTCCTGAACCTCTGG	62	n.a.	1.90
	rv	GGAGGCCATTCAAAACTTCA		n.a.	
**Circadian gene**					
				
*Dbp*	fw	AATGACCTTTGAACCTGATCCCGCT	175	7	1.93
	rv	GCTCCAGTACTTCTCATCCTTCTGT		7	

### Expression level of reference genes

**Figure 1:**
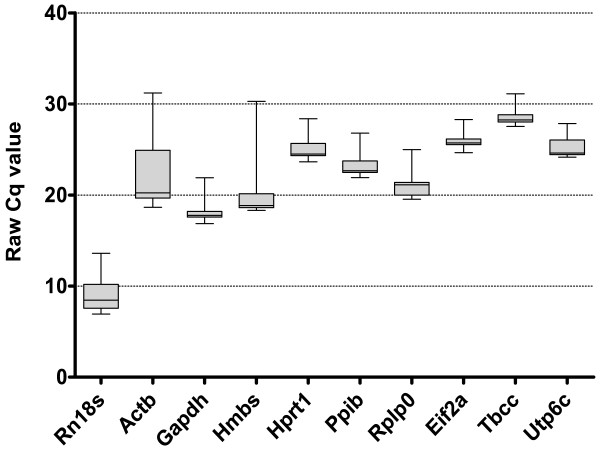
**Expression levels of candidate reference genes**. Expression is represented as quantification cycle values (Cq) obtained from qPCR. Variability is displayed as medians (line), 25^th ^percentile to 75^th ^percentile (box) and min to max (whiskers). Gene symbols are explained in Table 1. All experiments (three mouse strains, two tissues) were considered in this analysis.

Expression of measured reference genes is represented as raw quantification cycle (Cq) in Figure 1. *Rn18 s *shows highest expression in all samples with a mean Cq of 8.8. This is not surprising since *Rn18 s *represents the bulk of total RNA. The Cq values of other candidates were between Cq 18-29. *Tbcc *shows lowest expression in all samples with a mean Cq of 28.5. The largest variation across the studied 228 samples was observed in *Actb *and *Hmbs *(12 cycles) and the smallest for *Eif2a, Tbcc *and *Utp6c *(< 4 cycles).

### Search for optimal reference genes by geNorm

geNorm ranks reference genes according to their average expression stability value (M), from the most stable (lowest M value) to the least stable (highest M value). An important advantage of geNorm is that it provides the optimal number of reference genes required for accurate normalization. This number is based on the pairwise variation values (V_(n/n+1)_) [20].

We divided expression data into eight groups, according to the tissue (liver or adrenal) and mouse strain (C57BL/6JOlaHsd; 129Pas plus C57BL/6J; *Crem*KO in mixed strain or including all strains; Table 3). Groups A to F thus represent stably expressed reference genes of each tissue in each mouse strain. Groups G and H consider genetic differences between mouse strains because samples of all strains are joined for each tissue. The time of sacrifice (C_t_) cannot be included as a variable by geNorm analyses. The same groups have been applied as well in NormFinder analyses.

**Table 3:** Distribution of RT-qPCR data into groups

Mouse Strain	Tissue	Time Point [Ct]	Biological replicates	Dataset [num. of samples]
C57BL/6JOlaHsd	Liver	0	5	A [35]
		4	5	
		8	5	
		12	5	
		16	5	
		20	5	
		24	5	
	Adrenal gland	0	5	B [33]
		4	4	
		8	5	
		12	5	
		16	5	
		20	5	
		24	4	

129Pas plus C57Bl/6J	Liver	0	6	C [45]
*Crem *knock-out		4	6	
		8	7	
		12	6	
		16	6	
		20	7	
		24	7	
	Adrenal gland	0	3	D [30]
		4	4	
		8	4	
		12	5	
		16	4	
		20	5	
		24	5	

129Pas plus C57Bl/6J	Liver	0	8	E [51]
Wild type		4	7	
		8	7	
		12	7	
		16	7	
		20	8	
		24	7	
	Adrenal gland	0	5	F [34]
		4	5	
		8	4	
		12	5	
		16	5	
		20	5	
		24	5	

All strains	Liver	7 time points	131	G

All strains	Adrenal	7 time points	97	H

**Total number of samples**			**228**	

**Figure 2:**
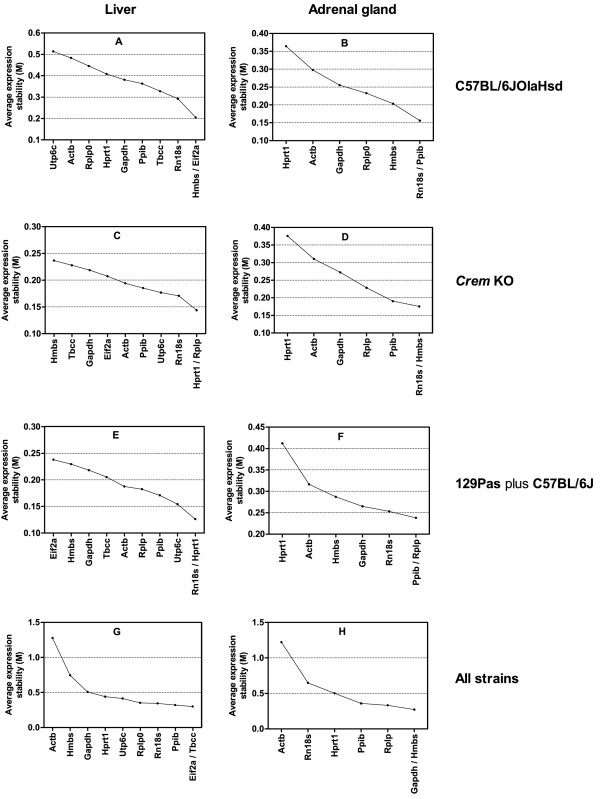
**Expression stabilities and ranking of reference genes in individual groups according to geNorm**. Groups A to F enable determination of reference genes for circadian experiments within separate tissues and mouse strains. Data is divided according to tissue type (liver and adrenal gland) and mouse strain. The remaining two groups (G and H) were used to identify reference genes when comparing different mouse strains. Figures are divided according to tissues (liver and andrenal glands) and mouse strain. Crem KO is on the 129Pas plus C57BL/6J background. Graphs G and H represent reference genes from joint data of all strains.

Figure 2 summarises the average expression stabilities and ranking of candidate reference genes in individual groups. Ranking is different for livers of different mouse strains. In 129Pas plus C57BL/6J and *Crem *KO, which has the *Crem *gene disrupted on the same mixed background, *Hprt1 *is always most stable, together with either *Rn18 s *(Figure 2E) or *Rplp0 *(Figure 2C). In the C57BL/6JOlaHsd strain, the most stable genes are *Eif2a *and *Hmbs *(Figure 2A), which were, interestingly, among the least stable in the mixed strain (Figure 2C,2E). In adrenal glands of individual mouse strains, the ranking is more consistent, with *Hprt1 *and *Actb *both ranking last and *Rn18 s *and *Ppib *always ranking among the top three (Figure 2B,2D,2F).

When evaluating reference genes within each strain, relatively small M values are observed (Figure 2A to 2F), indicating a greater degree of expression stability between samples. However, when genetic differences between mouse strains are included in evaluation, a larger degree of variation (larger M value) is observed (Figure 2G and 2H), which supports the notion that genetic variability importantly influences expression. In this case, *Actb *is the least stable in both liver and adrenal glands. It also displays a far greater M value compared to other genes, showing that it is indeed not a good gene for normalization in mouse tissues. *Eif2a *and *Tbcc *in the liver (Figure 2G) and *Gapdh *and *Hmbs *in adrenals (Figure 2H) are most stable if all strains are considered.

**Figure 3:**
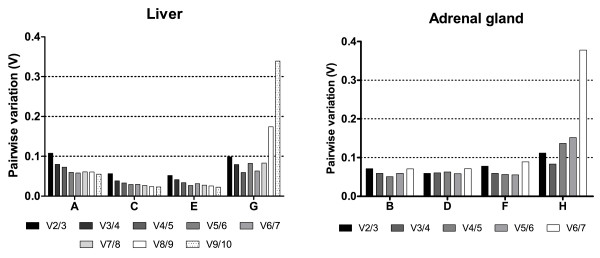
**Optimal number of reference genes used for normalization**. geNorm determination of the optimal number of reference genes based on the pairwise variation value (V**_n/n+1_**) that is calculated between two sequential normalization factors [20]. The optimal number of reference genes was calculated for liver and adrenal glands where samples from all mouse strains are included.

We also determined the optimal number of reference genes for normalization (Figure 3). In all groups (A - H), a pairwise variation value (V2/3) of less than 0.15 was determined, confirming that 2 stable reference genes might be sufficient for accurate normalization, as proposed by Vandesompele et al [20]. Addition of further genes in groups A to F did not influence the V value significantly. However, in groups G and H, addition of least stable genes did raise the V value above 0.15 (Figure 3).

### Determination of reference genes by NormFinder

NormFinder uses a model based approach to calculate gene stability value for either the most stable reference gene or the best combination of two genes. It has the advantage to allow estimation of variations between time points, which is crucial in circadian experiments. NormFinder gene stability values and rankings are shown in Table 4. In this analysis, samples in all groups (A - H) were divided further into 7 subgroups according to the time of scarification (time point).

**Table 4:** Stability value and ranking of reference genes based on NormFinder

	Circadian							
	C57BL/6JOlaHsd		129Pas plus C57Bl/6J-KO		129Pas plus C57Bl/6J-WT		Mouse strain	
Gene	liver	adrenal gland	liver	adrenal gland	liver	adrenal gland	liver	adrenal gland
Dataset	A	B	C	D	E	F	G	H
**Rn18s**	0.118	0.070	0.078	0.068	0.063	0.062	0.020	0.054
**Actb**	0.196	0.090	0.093	0.104	0.087	0.060	0.555	0.506
**Gapdh**	0.134	0.088	0.123	0.113	0.106	0.058	0.112	0.080
**Hmbs**	0.096*	0.058	0.114	0.080	0.095	0.063	0.309	0.088
**Hprt1**	0.150	0.156	0.062	0.166	0.058	0.187	0.024	0.199
**Ppib**	0.124	0.057*	0.090	0.062*	0.070	0.025*	0.028	0.028*
**Rplp0**	0.187	0.081	0.056*	0.099	0.077	0.073	0.021	0.064
**Eif2a**	0.127		0.112		0.106		0.017*	
**Tbcc**	0.124		0.107		0.081		0.021	
**Utp6c**	0.190		0.060		0.057*		0.027	

**Best two**	Ppib	Ppib	Hprt1	Rn18s	Hprt1	Gapdh	Rn18s	Rn18s
	Hmbs	Hmbs	Ppib	Ppib	Utp6c	Ppib	Eif2a	Ppib
	0.061	0.046	0.044	0.048	0.037	0.032	0.013	0.031

In groups A to F, data was grouped according to tissue, mouse strain and the time point. In groups G and H, data was grouped only according to tissue and time point. *-best candidate reference gene according to NormFinder. (Best two) - the best combination of two reference genes together with their stability value are calculated only if group identifiers (in our case time of sacrifice) are included in the analysis. "Best two" is not always equal to the first and second ranking genes, but represent the two genes with minimal combined inter-and intragroup expression variation [22].

Similarly to geNorm, NormFinder also showed differences in ranking of reference genes in different mouse strains. In liver samples of 129Pas plus C57BL/6J and *Crem *KO strains *Hprt1 *always ranks among the top two reference genes (Table 4C and 4E), however in the C57BL/6JOlaHsd strain it is among the least stable genes (Table 4A). The opposite is seen for *Hmbs*, which is most stable in the C57BL/6JOlaHsd strain (Table 4A) and among least stable in 129Pas plus C57BL/6J and *Crem *KO strains (Table 4C and 4E). When genetic differences between strains are included in the evaluation, *Actb *is the least stable gene (Table 4G), followed by *Hmbs *and *Gapdh*.

Ranking is again more consistent for adrenal glands. *Ppib *ranks as the most stable reference gene both in each individual mouse strain (Table 4B,D and 4F) and also irrespective of the mouse genetic background (Table 4H). *Hprt1 *is the least stable gene in adrenals of each investigated mouse strain (Table 4B, D and 4F), while *Actb *ranks last. Again, this result is obtained only if genetic differences between strains are taken into consideration (Table 4H).

In individual mouse strain groups (A - E), with the exception of group F, gene stability value for the best combination of two reference genes is lower than the value of the most stable reference gene (Table 4), suggesting that normalization on a single reference gene may not be sufficient.

### Normalization of biological data by reference genes of different stability

*Dbp *(D-box binding protein) is one of the most robust circadian genes in the liver, with the peak of expression between CT10-CT14 [26]. We monitored the *Dbp *hepatic expression (Cq values) in mouse strains C57BL/6JOlaHsd, 129Pas plus C57BL/6J and *Crem *KO. To test the role of reference gene selection on interpretation of the hepatic *Dbp *expression, we applied different normalization procedures: with a) *Actb *which is commonly used for normalization, but was determined as the least stable gene by geNorm and NormFinder; b) the average of the two most stable reference genes according to geNorm (*Eif2a *and *Tbcc*) and c) the average of the two most stable reference genes according to NormFinder (*Rn18 s *and *Eif2a*).

**Figure 4:**
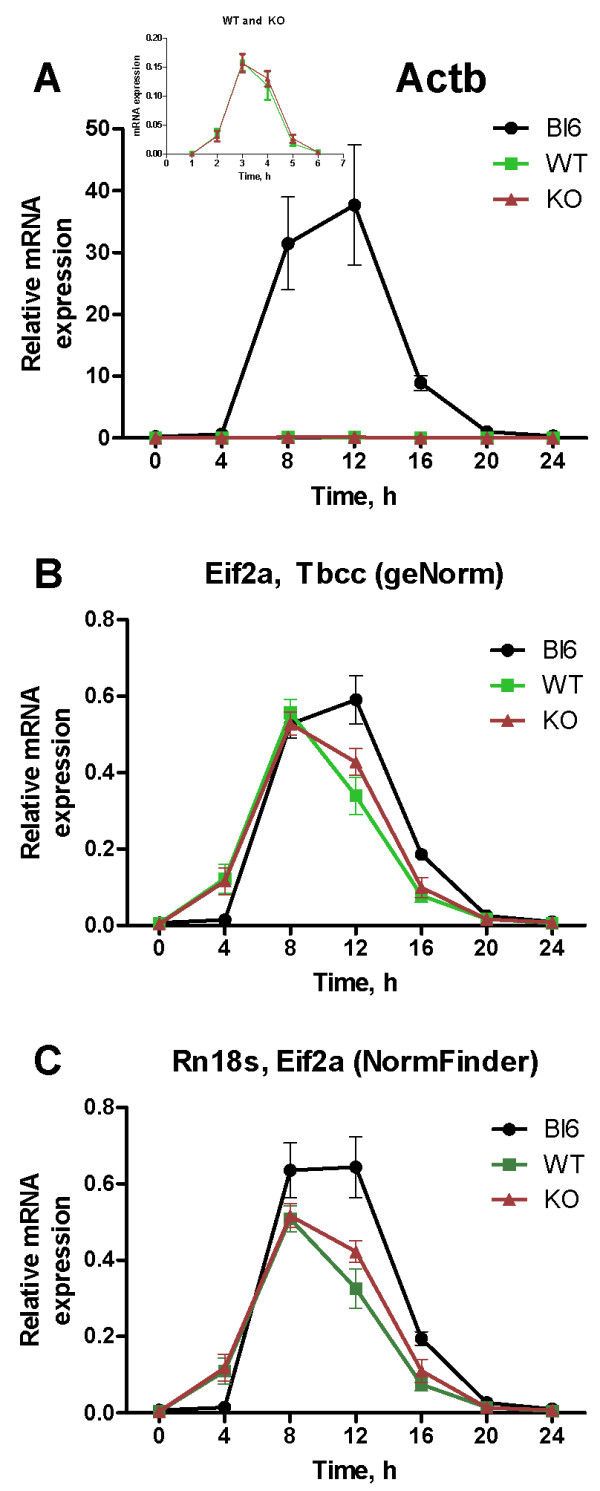
**Liver expression profile of the mouse circadian gene *Dbp***. The ***Dbp ***gene with known circadian transcription in mouse liver was normalized with an unstable reference gene ***Actb ***(A) or with the two most stable reference genes as calculated by geNorm (B) or NormFinder (C). Due to low values not seen in panel A, an insert represents the expression profile of ***Dbp ***in 129Pas plus C57BL/6J and ***Crem ***KO mouse strains.

Figure 4 shows that normalization to *Actb *inserts bias into the data. A seemingly large difference in *Dbp *expression is detected between the C57BL/6JOlaHsd and the 129Pas plus C57BL/6J and *Crem *KO strains (Figure 4A). This leads to a false conclusion that *Dbp *has a very high expression in the C57BL/6JOlaHsd strain, with almost none in the 129Pas plus C57BL/6J and *Crem *KO strains. However, when normalization is carried out with the most stable reference genes determined by either geNorm or NormFinder, the difference is greatly reduced (Figure 4B and 4C). In this case, *Dbp *is equally expressed in both mouse strains, which can be expected for a gene with a high expression level and robust circadian rhythm [27].

## Discussion

qPCR is a method of choice for quantitative gene expression analysis. Due to its high sensitivity, normalization with stable reference genes is important for accurate analysis of the biological variation in the data [18,23,28]. The selection of appropriate reference genes is, however, far from trivial. It has been shown that application of non-validated reference genes can lead to inaccurate data interpretation [23,29,30].

Understanding the tissue-specific circadian behaviour of genes and proteins is often required in drug-treated mouse models, including knockout models from different mouse strains. In recent years, the circadian aspects of metabolism and drug detoxification became more important for proper understanding of physiology, pathophysiology, drug metabolism, etc. [5,31-34]. Even though a number of studies discuss the selection of reference genes [35-39] no study discussed circadian experiments and only one past study evaluated different mouse strains [40]. The majority of circadian studies still perform normalization using a single reference gene [41-44].

We evaluate ten candidate reference genes for their expression stability in a circadian experiment with mouse liver and adrenal glands in three mouse strains (C57BL/6JOlaHsd, 129Pas plus C57BL/6J and *Crem *KO). Seven genes from our study have been evaluated previously under a variety of experimental conditions [20,45,46]. Three additional genes (*Eif2a, Tbcc *and *Utp6c*) were selected by RefGene as the most stably expressed after the meta analysis of liver Affymetrix expression profile [25]. Publicly available microarray data have been successfully used before for reference gene selection [47,48]. We show that irrespective of the analysis applied (geNorm or NormFinder), different mouse strains show different rankings of reference genes. *Utp6c *was indicated as unstable reference gene in C57BL/6JOlaHsd livers by both programs (Figure 2A, Table 4A), whereas in the 129Pas plus C57BL/6J and *Crem *KO it ranked among the top three (Figure 2C and 2E; Table 4C, E).

When comparing gene expression between different mouse strains, three reference genes from the Affymetrix meta analyses ranked among the most stable, with *Eif2a *being first by both programs (Figure 2G and Table 4G). This indicates that RefGenes tool is useful in narrowing down the number of candidate reference genes, especially when comparing mouse strains.

Differences in ranking of reference genes were observed not only between mouse strains, but also between tissues, as confirmed by both programs. Genes most stable in livers of 129Pas plus C57BL/6J and *Crem *KO strains ranked among the least stable in C57BL/6JOlaHsd mice (Figure 2 and Table 4). Adrenals show similar ranking between strains. *Ppib *is usually the most stable and *Hprt1 *the least stable gene in all strains by both programs. Our study shows that reference genes suitable for one mouse strain should not automatically be used for normalization in another strain. The same applies for different tissues.

The average expression stability value M determined by geNorm shows little variability in gene expression between samples taking into account circadian effect both in liver and adrenals within each strain. This is in line with the pairwise variation value (V2/3), which is well below the 0.15 threshold value set by Vandensompele [20], indicating that normalization of target genes with a combination of the two best genes is sufficient. The M value of reference genes was substantially higher when searching for stable genes between mouse stains. Here *Actb *was identified as the least stable in both liver and adrenals (Figure 2G,2H and Table 4G, H). Similar results were obtained when comparing human tissue samples [20].

To test whether selection of the least stable reference gene (*Actb*) affects normalization when compared to normalization with the two most stable genes selected by geNorm and NormFinder, a known and robust liver circadian gene *Dbp *has been normalized to three factors (Figure 4). Normalization to *Actb *leads to a large difference in the expression pattern between the C57BL/6JOlaHsd strain and the 129Pas plus C57BL/6J and *Crem *KO strains (Figure 4A). This can lead to a false impression that expression of *Dbp *is substantially higher in C57BL/6JOlaHsd. This difference, however, almost disappears when normalisation is performed on the two most stable genes *Eif2a *and *Tbcc *(geNorm) or *Eif2a *and *Rn18 s *(NormFinder) (Figure 4B and 4C). Even though the programs did not select identical reference genes, a similar *Dbp *expression profile was obtained in both cases. However, the ability of NormFinder to distinguish between different time points provides an advantage over geNorm when sufficient samples and genes are evaluated [22], as is the case in circadian experiments.

## Conclusions

In this study we investigated the most reliable reference genes for normalization of circadian studies within or between mouse strains in livers and adrenal glands. The study is unique in its analysis of 3 mouse strains, 2 tissues and circadian sampling and in the magnitude of samples and genes tested (10 candidate reference genes in 228 samples by two programs). We show that differences in the reference genes exist between mouse strains as well as between tissues of the same strain. We also show that selection of a reference gene that appears stable in each mouse strain separately, can lead to interpretation errors, when used for normalization in different mouse stains. We identified altrernative reference genes that are stable in mouse strain comparisons.

## Methods

### Animals

54 wild type (129Pas plus C57BL/6J) and 45 *Crem *knock-out (*Crem *KO) mice of the mixed strain and 35 mice of the inbred strain (C57BL/6JOlaHsd) were used. Animals had free access to food (Harland Tekland 2916) and water and were maintained under a 12:12 h light cycle (light on at 7:00 am, light off at 7:00 pm). The experiment was approved by the Veterinary Administration of the Republic of Slovenia (license number 34401-9/2008/4) and was conducted in accordance with the European Convention for the protection of vertebrate animals used for experimental and other scientific purposes (ETS 123) as well as in accordance with National Institutes of Health guidelines for work with laboratory animals.

### Tissue samples

Mice were sacrificed with cervical dislocation under dim red light every 4 h over a 24 h period starting on the second day after being transferred to dark: dark (DD) conditions. Immediately after they were sacrificed, liver and adrenal glands were excised, snap frozen in liquid N_2 _and stored at -80°C for subsequent analysis. 96 liver and 58 adrenal glands from the mixed strain and 35 liver and 34 adrenal glands from the inbred strain were used for cDNA synthesis. The lower number of adrenal glands is due to insufficient amount of material or due to small RNA isolation yield. RNA was isolated from 30 mg of liver tissue and one adrenal gland per animal.

### RNA extraction and cDNA preparation

Liver and adrenal gland samples were homogenized in 1000 μl and 500 μl of TRI reagent (Sigma) respectively and total RNA was isolated according to the manufactures instructions. RNA from liver samples of the inbred strain was isolated using QuickGene-810 (FujiFilm) according to manufacturer's instructions. RNA quantity and quality were assessed with NanoDrop and Agilent 2100 Bioanalyzer instruments. DNAse treatment was performed on all samples using DNAse I (Roche Applied Bioscience) according to the manufacturer's instructions. cDNA synthesis was carried out using SuperScript III reverse transcriptase (Invitrogen). 3 μg of liver RNA was mixed together with 20 μl of reverse transcriptase master mix which contained 8 μl of 5 × first strand buffer, 2 μl of 100 mM DTT, 2 μl of 10 mM dNTP mix, 1 μl of random primers (Promega 500 ng/μl), 0.75 μl of SuperScript III (200 U/μl), 0.75 μl of RNAse OUT (Invitrogen) and 5.5 μl of RNAse free water in a final volume of 40 μl. 1 μg of adrenal gland RNA was mixed together with 10 μl of reverse transcriptase master mix which contained 5 μl of 5x first strand buffer, 1.25 μl of 100 mM DTT, 1.25 μl of 10 mM dNTP mix, 0.65 μl of random primers (Promega 500 ng/μl), 0.5 μl of SuperScript III (200 U/μl), 0.5 μl of RNAse OUT (Invitrogen) and 0.85 μl of RNAse free water in a final volume of 25 μl. The reaction mixtures were incubated at 25°C for 5 minutes, 50°C for 60 minutes and 70°C for 10 minutes.

### Primer design

Wherever possible, intron spanning primers for ten candidate reference genes were designed based on publicly available sequences (Table 2). Genes chosen belong to different functional classes, which reduces the chance of co-regulation. Primer specificity and amplification efficiency were also validated empirically with melting curve and standard curve analysis of a six fold dilution series.

### Quantitative qPCR

Real time quantitative PCR was performed in a 384 well format on LightCycler 480 (Roche Applied Science) using LightCycler 480 SYBR Green I Master (Roche Applied Science). The PCR reaction consisted of 2.5 μl of SYBR Green I Master, 1.15 μl of RNAse free water, 0.6 μl of 300 nM primer mix and 0.75 μl of cDNA in a total volume of 5 μl. Three technical replicates were performed for each sample. Cycling conditions were as follows: 10 min at 95°C followed by 40 rounds of 10 s at 95°C, 20 s at 60°C and 20 s at 72 °C. Melting curve analysis for determining the dissociation of PCR products was performed from 65°C to 95°C.

### Analysis of expression stability

Expression stabilities of selected reference genes were evaluated by two publicly available programs, *geNorm *VBA and *NormFinder *applets for Microsoft Excel. *geNorm *calculates the stability of selected reference genes according to the similarity of their expression profile by pair-wise comparison and calculates M value, where the gene with the highest value is the least stable one. All calculations were performed on quantities, which were transformed from Cq values based on gene specific efficiencies [20]. *NormFinder *also requires quantities input, but calculates a gene-stability value with a mathematical model based on separate analysis of the sample subgroups and estimation of both intra-and intergroup variation in expression levels [22]. Relative quantities (Q) needed for the input were calculated via the delta-Ct method with the formula Q = (E)^ΔCq^. ΔCq equals Cq of the sample with the lowest Cq value (highest abundance) minus Cq of a sample. Efficiency (E) corrected relative amount were calculated. Normalization factors were calculated based on geometric averaging of the most stable reference genes. Normalization was carried out by dividing relative quantities with the normalization factor [20].

## Authors' contributions

RK carried out qPCR and data analysis and wrote the manuscript. JA and MG participated in the planning, sampling and data analysis. MP and GM are responsible for animal experiments and participated in sampling, design of the study and provided useful discussions. MF participated in study design, data analysis and provided useful discussion.DR supervised the study, participated in study design and coordination. All authors read and approved the final manuscript.
